# Weight, height, waist circumference: association with knee osteoarthritis findings from the osteoarthritis initiative

**DOI:** 10.1097/PR9.0000000000001187

**Published:** 2024-09-20

**Authors:** Lisa H. Antoine, Kristen Allen Watts, Deanna D. Rumble, Taylor Buchanan, Andrew Sims, Burel R. Goodin

**Affiliations:** a Department of Psychology, University of Alabama at Birmingham, Birmingham, AL, USA; b Heersink School of Medicine, University of Alabama at Birmingham, Birmingham, AL, USA; c Department of Psychology and Counseling, University of Central Arkansas, Conway, AR, USA; d Department of Family and Community Medicine, University of Alabama at Birmingham, Birmingham, AL, USA; e Department of Medicine, University of Mississippi Medical Center, Jackson, MS, USA; f Washington University Pain Center, Department of Anesthesiology, Washington University, St. Louis, MO, USA

**Keywords:** Weight, Height, Waist circumference, Knee osteoarthritis

## Abstract

**Introduction:**

Global prevalence of knee osteoarthritis is more than 300 million. Uncontrollable risk factors include age, sex, and height. Controllable risk factors include trauma, weight, and waist circumference.

**Objectives:**

Our goal was to determine the association between knee osteoarthritis and anthropometric measures that include weight, height, and waist circumference.

**Methods:**

Using 4,602 participants (45–79 years) from the Osteoarthritis Initiative, we analyzed the association between knee osteoarthritis and anthropometry collectively and by sex. We calculated female and male tertiles (3 groups) for anthropometry.

**Results:**

Anthropometric measures were correlated with knee osteoarthritis (*P* ≤ 0.05) except the correlation between height and activities and height and quality of life. When comparing female weight tertiles, there were associations (*P*'s < 0.001) between knee osteoarthritis and weight, but when comparing male weight tertiles, these associations were primarily between the lowest weight and highest weight groups. There were significant associations between knee osteoarthritis and height among female tertiles, with no differences among male tertiles. There were knee osteoarthritis/waist circumference tertile associations (*P*'s < 0.001) for the lowest and highest waist circumference groups.

**Conclusion:**

Higher weight in female participants was a stronger predictor of increases in knee osteoarthritis discomforts when compared to waist circumference, while weight and waist circumference were almost equivalent in predicting increases in knee osteoarthritis for male participants. Height did not predict increases in knee osteoarthritis with the exception of female symptoms and quality of life. Quality of life for both sexes was the most unfavorable with female participants reporting a more unfavorable quality of life than male participants.

## Abstract 2

Supplemental Digital Content is Available in the Text.

Higher weight/knee osteoarthritis association was greater than larger waist circumference/knee osteoarthritis association for female participants, while these associations were almost equivalent for male participants.

## 1. Introduction

In 1990, globally knee osteoarthritis (KOA) cases were 163,910,000.^15^ After almost 3 decades, KOA rose to 364,580,000 cases^15^ with prevalence 69% greater for women.^3^ Knee osteoarthritis cases are expected to rise. Uncontrollable risk factors for KOA include age, sex, race,^7^ and height. Controllable risk factors include trauma, muscle weakness, weight,^7^ and waist circumference. In this study, we investigate the impact of weight, height, and waist circumference (anthropometric measures) on KOA.

Researchers found an association between higher weight and KOA.^1,25^ Twenty-three studies highlighted harmful effects of weight gain (≥5%) on knee pain, stiffness, function, and cartilage damage.^23^ With no remedy for KOA, it is important to identify relief options. To mitigate KOA discomforts, weight loss combined with diet and exercise was recommended in clinical practice guidelines.^14^ Even after weight loss, researchers did not notice a cartilage thickness change.^11^ The effect of weight loss on KOA revealed that lower baseline body mass index (BMI) adults experienced a relief benefit when compared to higher baseline BMI adults.^17^ Even with weight loss, KOA damage may linger causing clinicians to recommend total knee replacement (TKA). Higher weight categories were associated with an increased risk of TKA revisions for infection.^22^

Even though height contributions to KOA were ambiguous, taller women seemed to have greater odds of KOA development after BMI adjustment.^1^ Researchers found a correlation between height and KOA development risk,^10^ an association between height and KOA radiographic severity,^24^ and evidence of the harmful effects of increased height on osteoarthritis risk.^10^ When considering TKA revisions for noninfection, there was an association with the tallest height category.^22^

When comparing first and fourth quartiles (4 groups) for waist circumference and its association with KOA, larger waist circumferences had higher odds of KOA.^1^ Using gender-specific waist circumference tertiles (3 groups) nested into BMI tertiles, middle and high waist circumference in the middle BMI tertile were more likely to have KOA, and in the highest BMI tertile, the highest waist circumference tertile was more likely to have KOA compared to the lowest waist circumference tertile.^9^ Knee osteoarthritis was associated with higher waist circumference regardless of weight.^4,16^ Research suggested that larger waist circumferences are associated with KOA or the risk of KOA.^13,25^

Based on previous studies, it is not clear which anthropometric measure—weight, height, or waist circumference—affects KOA the most. We are not aware of any study that analyzes the impact of all 3 anthropometric measures on KOA using the same cohort collectively and by sex. We fill that gap by investigating which of these anthropometric measures was the strongest predictor of KOA. We calculated the overall association of each measure on KOA. Then we analyzed these associations by sex. After categorizing each measure by sex into tertiles with increasing weight, height, and waist circumference, we determined whether groups in higher tertiles experienced greater knee discomforts. We expected participants in higher weight and waist circumference groups would experience greater knee discomforts, when comparing the sexes, and when categorizing each sex into tertiles. We did not expect height to be associated with KOA to the extent that weight and waist circumference would be associated with KOA.

## 2. Methods

### 2.1. Data

Our group used data from the Osteoarthritis Initiative (OAI) database. Osteoarthritis initiative was a 10-year, data collection effort with an enrollment period from February 2004 to May 2006.^8^ Data collection sites included Ohio State University, University of Maryland School of Medicine, University of Pittsburgh School of Medicine, Brown University School of Medicine, and Memorial Hospital of Rhode Island. University of California, San Francisco School of Medicine served as the coordination center. The OAI database includes clinical evaluation data, radiological images, and a biospecimen repository.

### 2.2. Participants

Osteoarthritis initiative excluded adults younger than 45 years and older than 79 years. Adults with rheumatoid arthritis (RA), inflammatory arthritis, and past or present users of RA-prescription medications were excluded. Adults likely to experience severe joint space narrowing during the study, those with total knee replacement on both knees, and those planning total knee replacement on both knees in the next 3 years were excluded. Women with a positive pregnancy test were excluded. Any adult unable to provide a blood sample for any reason was excluded. Adults using a walking aid (other than a single straight cane) more than 50% of the time were excluded. Adults with diseases that prohibit participation in a 4-year study were excluded and those who may relocate from the clinic area prior to 3 years. Any adult participating in a double-blind randomized controlled trial was excluded. Adults unable to sign informed consent were excluded.

After exclusions, the OAI cohort included 4,796 women and men ages 45 to 79 years with clinical symptomatic KOA or at risk for developing KOA. Using OAI data, the first objective was to analyze the collective association of anthropometric measures—weight, height, and waist circumference with KOA. The second objective was to analyze by sex KOA associated with categorized anthropometric measures. Therefore, we excluded adults with missing weight, height, and waist circumference data. After exclusions, there were 4,602 eligible participants.

### 2.3. Measures

#### 2.3.1. Demographics

Data extracted from the OAI database included age, sex, racial background, education, employment status, income, and marital status. Refer to the appendix, http://links.lww.com/PR9/A245 for variable names and values for demographic data.

#### 2.3.2. Anthropometry

Data extracted from the OAI database included weight (kg), height (mm), and waist circumference (cm). Refer to the appendix, http://links.lww.com/PR9/A245 for variable names and values for anthropometric data.

#### 2.3.3. Pain

Data extracted from the OAI database included right knee and left knee Western Ontario and McMaster Universities Osteoarthritis Index (WOMAC) pain, stiffness, disability, and total scores. WOMAC pain scores range from 0 to 20 with higher scores equal to greater pain. WOMAC stiffness scores range from 0 to 8 with higher scores equal to greater stiffness. WOMAC disability scores range from 0 to 68 with higher scores equal to greater disability. WOMAC total scores are a sum of pain, stiffness, and disability scores and range from 0 to 96. WOMAC pain scores are dimensionless. Refer to the appendix, http://links.lww.com/PR9/A245 for variable names and values for WOMAC data.

Data extracted from the OAI database included Knee Outcomes of Osteoarthritis Scale (KOOS) pain and symptoms scores for the right and left knees, function, sports, and recreational score, and quality of life score. Knee Outcomes of Osteoarthritis Scale pain, symptoms, function, sports, and recreational, and quality of life scores range from 0 to 100 with higher scores equal to less pain and symptoms, greater knee functionality, and greater quality of life. Knee Outcomes of Osteoarthritis Scale pain scores are dimensionless. Refer to the appendix, http://links.lww.com/PR9/A245 for variable names and values for KOOS data.

### 2.4. Statistical analysis

Using IBM SPSS 28.0,^6^ our group performed statistical analysis on demographic, anthropometry, and pain measures from the OAI database (clinical data version 0.2.3). We checked for duplicate participant entries and missing values.

Descriptive statistics and one-way ANOVA were used to calculate means, standard deviations, *P*-value for continuous variables, and χ^2^ for categorical variables. The test for linearity was used to calculate the linearity and deviation relationship between anthropometry and pain measures. Each measure was assessed for normality. The measures were normally distributed. SPSS frequency statistics were used to determine percentage distributions for the categorical variables.

Anthropometry and pain measures were compared among the entire sample and by sex. To determine the relationship between anthropometric and pain measures for the sample, bivariate correlations were used to calculate Pearson correlation coefficients with a two-tailed significance of 0.05. We calculated tertiles (2 points that divided the participants into approximately 3 numerically equal groups) to separate each anthropometric measure into thirds to observe a pattern of increased pain with increased weight, height, and waist circumference by sex. Univariate analysis of variance was used to investigate by sex the effects of 3 weight categories, 3 height categories, and 3 waist circumference categories on WOMAC and KOOS pain scores and to compare pain scores between categories for each anthropometric measure.

Three categories for female weight included (1) 67.30 kg or less, (2) between 67.30 kg and 81.50 kg, and (3) 81.50 kg and greater. Three categories for female height included (1) 1597.50 mm or less, (2) between 1597.50 mm and 1649.50 mm, and (3) greater than 1649.50 mm. Three categories for female waist circumference included (1) 95.65 cm or less, (2) between 95.65 cm and 107.50 cm, and (3) 107.50 cm and greater.

Three categories for male weight included (1) 82.30 kg or less, (2) between 82.30 kg and 95.00 kg, and (3) 95.00 kg and greater. Three categories for male height included (1) 1734.50 mm or less, (2) between 1734.50 mm and 1791.00 mm, and (3) greater than 1791.00 mm. Three categories for male waist circumference included (1) 97.50 cm or less, (2) between 97.50 cm and 107.80 cm, and (3) 107.80 cm and greater.

Using these anthropometric categories, we applied the linear regression model for females and then for males to predict whether increases in weight, height, and waist circumference result in increases in WOMAC scores and decreases in KOOS scores.

## 3. Results

### 3.1. Participants

Out of 4,602 participants, 18.6% were Black or African Americans and 78.7% were White or Caucasian. 58.6% were female. More than 58% graduated college. Greater than 60% worked for pay. The annual income for 55.6% of the participants was greater than or equal to $50,000. 66% were married. See Table 1 for demographics.

**Table 1:** Demographics and anthropometry—overall and by sex.

	Total sample (n = 4602)	Female (n = 2698)	Male (n = 1904)	*P* or χ^2^
Age, mean ± SD	61.33 ± 9.16	61.48 ± 8.92	61.12 ± 9.49	0.181
Racial background, n (%)				<0.001
Black or African American	854 (18.6%)	585 (21.7%)	269 (14.1%)	
White or Caucasian	3620 (78.7%)	2034 (75.4%)	1586 (83.3%)	
Asian	43 (0.9%)	30 (1.1%)	13 (0.7%)	
Other	80 (1.7%)	47 (1.7%)	33 (1.7%)	
Education, n (%)				<0.001
Less than high school	162 (3.5%)	98 (3.6%)	64 (3.4%)	
High school degree	591 (12.8%)	421 (15.6%)	170 (8.9%)	
Some college	1103 (24.0%)	772 (28.6%)	331 (17.4%)	
Four-year college degree	957 (20.8%)	505 (18.7%)	452 (23.7%)	
Some graduate school	383 (8.3%)	199 (7.4%)	184 (9.7%)	
Graduate degree	1366 (29.7%)	682 (25.3%)	684 (35.9%)	
Income, n (%)				<0.001
Less than $10,000	155 (3.4%)	101 (3.7%)	54 (2.8%)	
$10,000 to <$25,000	443 (9.6%)	325 (12.0%)	118 (6.2%)	
$25,000 to <$50,000	1096 (23.8)	736 (27.3%)	360 (18.9%)	
$50,000 to <$100,000	1550 (33.7%)	866 (32.1%)	684 (35.9%)	
$100,000 or greater	1006 (21.9%)	428 (15.9%)	578 (30.4%)	
Employment status, n (%)				<0.001
Working	2792 (60.7%)	1546 (57.3%)	1246 (65.4%)	
Not working	1787 (38.9)	1138 (42.2%)	649 (34.2%)	
Marital status, n (%)				<0.001
Married	3037 (66%)	1542 (57.2%)	1495 (78.5%)	
Not married	1524 (33.1%)	1132 (41.9%)	392 (20.6%)	
Anthropometry				
Weight (kg), mean ± SD	81.34 ± 16.18	75.37 ± 14.65	89.80 ± 14.38	<0.001
Height (mm), mean ± SD	1681.26 ± 92.40	1623.94 ± 60.50	1762.48 ± 64.78	0.000
Waist circumference (cm), mean ± SD	102.43 ± 12.91	101.81 ± 13.76	103.30 ± 11.55	<0.001

n, sample size; *P* or χ^2^, probability for continuous and categorical variables, respectively; SD, standard deviation.

Participants were divided into tertiles (roughly thirds) and categorized by sex (female or male) for weight, height, and waist circumference. See Tables 2 and 3 for female and male, respectively, weight, height, and waist circumference approximate tertile categories. Average weight and waist circumference for females were 75.37 kg and 101.81 cm, respectively. Average height for males was 1762.48 mm. See Table 1 for anthropometry overall and by sex.

**Table 2:** Female categories into tertiles for weight, height, and waist circumference.

	n	Percent
Weight		
67.30 kg or less	902	33.4
Between 67.30 kg and 81.50 kg	905	33.5
81.50 kg or greater	891	33.0
Height		
1597.50 mm or less	904	33.5
Between 1597.50 mm and 1649.50 mm	904	33.5
1649.50 mm or greater	890	33.0
Waist circumference		
95.65 cm or less	902	33.4
Between 95.65 cm and 107.50 cm	909	33.7
107.50 cm or greater	887	32.9

Tertile is separated into approximately 3 equivalent groups.

cm, centimeters; kg, kilograms; mm, millimeters; n, sample size.

**Table 3:** Male categories separated into tertiles for weight, height, and waist circumference.

	n	Percent
Weight		
82.30 kg or less	634	33.3
Between 82.30 kg and 95.00 kg	635	33.4
95.00 kg or greater	635	33.4
Height		
1734.50 mm or less	636	33.4
Between 1734.50 mm and 1791.00 mm	639	33.6
1791.00 mm or greater	629	33.0
Waist circumference		
97.50 cm or less	638	33.5
Between 97.50 cm and 107.80 cm	636	33.4
107.80 cm or greater	630	33.1

Tertile is separated into approximately 3 equivalent groups.

cm, centimeters; kg, kilograms; mm, millimeters; n, sample size.

### 3.2. Pain

Mean WOMAC scores ranged from 1.44 ± 1.66 (stiffness left knee) to 8.29 ± 11.39 (disability left knee) and mean KOOS scores ranged from 66.36 ± 22.44 (quality of life) to 86.63 ± 15.82 (symptoms left knee).

Weight, height, and waist circumference were significantly correlated with all WOMAC scores (*P* ≤ 0.001) and all KOOS scores (*P* ≤ 0.05) with the exception of the correlation between height and function, sports, and recreational activities (*P* = 0.134) and height and quality of life (*P* = 0.107). Weight and height predicted all KOOS and WOMAC scores. Waist circumference predicted all KOOS scores (*P* ≤ 0.05), but only 50% of WOMAC (right knee pain, left knee pain and stiffness) scores (*P* ≤ 0.05).

#### 3.2.1. Female

Mean WOMAC scores ranged from 1.55 ± 1.72 (stiffness left knee) to 9.22 ± 11.35 (disability right knee) and mean KOOS scores ranged from 66.10 ± 22.58 (quality of life) to 85.61 ± 16.26 (symptoms left knee).

##### 3.2.1.1. Weight

**Figure 1.**
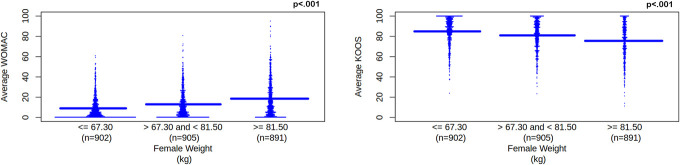
Average WOMAC and KOOS scores for female weight category. Female weight category is separated into tertiles. Weight unit is kg, which represents kilogram. WOMAC and KOOS scores are unitless. WOMAC represents Western Ontario and McMaster Universities Osteoarthritis Index. As weight increases, increasing WOMAC scores (left pane) represent worsening knee pain, disability, function, and total for left and right knees. KOOS represents Knee Outcomes of Osteoarthritis Scale. As weight increases, decreasing KOOS scores (right pane) represent worsening knee pain and symptoms for left and right knees; worsening function, recreation, and daily activities; and worsening quality of life. Solid blue line represents the mean for each tertile. KOOS, Knee Outcomes of Osteoarthritis Scale; WOMAC, Western Ontario and McMaster Universities Osteoarthritis Index.

Weight significantly correlated with all WOMAC and KOOS scores (*P*'s < 0.001). There were significant differences (*P* < 0.001) for WOMAC and KOOS scores among weight categories. We used a linear regression model that suggested as weight increased, the average WOMAC right and left knee totals (pain, stiffness, and disability) increased, β = 1.587, *P* < 0.001, 95% CI [1.368, 1.806] and average KOOS (left and right knee pain and symptoms, function, sports, and recreational activities, and quality of life) decreased, β = −4.634, *P* < 0.001, 95% CI [−5.482, −3.787]. See Figure 1. Adjusting for height, as weight increased average WOMAC scores increased, for weight β = 1.671, *P* < 0.001, 95% CI [1.445, 1.898] and for height β = −0.371, *P* = 0.006, 95% CI [−0.543, −0.090] and average KOOS scores decreased, for weight β = −4.718, *P* < 0.001, 95% CI [−5.594, −3.842] and for height β = −0.540, *P* = 0.456, 95% CI [−0.540, 1.203].

##### 3.2.1.2. Height

**Figure 2.**
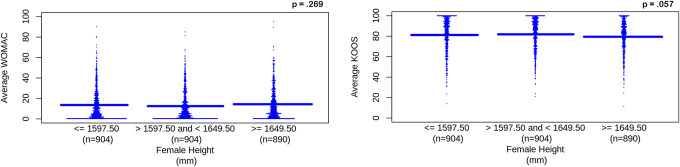
Average WOMAC and KOOS scores for female height category. Female height category is separated into tertiles. Height unit is mm, which represents millimeters. WOMAC and KOOS scores are unitless. WOMAC represents Western Ontario and McMaster Universities Osteoarthritis Index. As height increases, increasing WOMAC scores (left pane) represent worsening knee pain, disability, function, and total for left and right knees. KOOS represents Knee Outcomes of Osteoarthritis Scale. As height increases, decreasing KOOS scores (right pane) represent worsening knee pain and symptoms for left and right knees; worsening function, recreation, and daily activities; and worsening quality of life. Solid blue line represents the mean for each tertile. KOOS, Knee Outcomes of Osteoarthritis Scale; WOMAC, Western Ontario and McMaster Universities Osteoarthritis Index.

Height significantly correlated with right and left knee KOOS symptoms and quality of life (*P*'s < 0.05). There did not appear to be any clinical significance for height tertiles between 1649.50 mm or greater and 1597.50 mm to 1649.50 mm (WOMAC right knee stiffness and disability and all KOOS scores with the exception of function, sports, and recreational activities), between 1649.50 mm or greater and 1597.50 mm or less (KOOS symptoms and quality of life), and between 1597.50 mm or less and 1597.50 mm to 1649.50 mm (WOMAC left knee pain). Using linear regression, an increase in height did not appear to significantly change average WOMAC and average KOOS scores (*P* > 0.05). See Figure 2.

##### 3.2.1.3. Waist circumference

**Figure 3.**
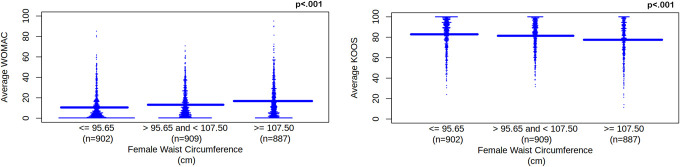
Average WOMAC and KOOS scores for female waist circumference category. Female waist circumference category is separated into tertiles. Waist circumference unit is cm, which represents centimeters. WOMAC and KOOS scores are unitless. WOMAC represents Western Ontario and McMaster Universities Osteoarthritis Index. As waist circumference increases, increasing WOMAC scores (left pane) represent worsening knee pain, disability, function, and total for left and right knees. KOOS represents Knee Outcomes of Osteoarthritis Scale. As waist circumference increases, decreasing KOOS scores (right pane) represent worsening knee pain and symptoms for left and right knees; worsening function, recreation, and daily activities; and worsening quality of life. KOOS, Knee Outcomes of Osteoarthritis Scale; WOMAC, Western Ontario and McMaster Universities Osteoarthritis Index.

Waist circumference significantly correlated with all WOMAC and KOOS scores (*P*'s < 0.001). There were significant differences (*P*'s ≤ 0.05) among waist circumference categories for WOMAC and KOOS scores with the exception of between 95.65 cm or less and 95.65 cm to 107.50 cm (KOOS right knee pain and KOOS symptoms). We used a linear regression model that suggested as waist circumference increased average WOMAC increased, β = 3.121, *P* < 0.001, 95% CI [2.451, 3.791] and average KOOS decreased, β = −2.617, *P* < 0.001, 95% CI [−3.482, −1.752]. See Figure 3.

#### 3.2.2. Male

Western Ontario and McMaster Universities Osteoarthritis Index scores ranged from 1.27 ± 1.56 (stiffness left knee) to 7.11 ± 10. 36 (disability left knee) and average KOOS scores ranged from 66.73 ± 22.23 (quality of life) to 88.07 ± 15.06 (symptoms left knee).

##### 3.2.2.1. Weight

Weight significantly correlated with WOMAC and KOOS scores (*P*'s < 0.001).

**Figure 4.**
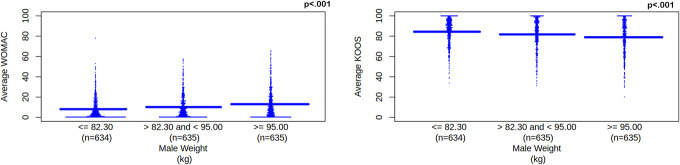
Average WOMAC and KOOS scores for male weight category. Solid blue line represents the mean for each tertile. Male weight category is separated into tertiles. Weight unit is kg, which represents kilogram. WOMAC and KOOS scores are unitless. WOMAC represents Western Ontario and McMaster Universities Osteoarthritis Index. As weight increases, increasing WOMAC scores (left pane) represent worsening knee pain, disability, function, and total for left and right knees. KOOS represents Knee Outcomes of Osteoarthritis Scale. As weight increases, decreasing KOOS scores (right pane) represent worsening knee pain and symptoms for left and right knees; worsening function, recreation, and daily activities; and worsening quality of life. Solid blue line represents the mean for each tertile. KOOS, Knee Outcomes of Osteoarthritis Scale; WOMAC, Western Ontario and McMaster Universities Osteoarthritis Index.

There were significant differences (*P* < 0.05) for WOMAC and KOOS scores among weight categories except when comparing 82.30 kg or less and between 82.30 kg and 95.00 kg (WOMAC right knee pain, stiffness, and disability and KOOS right knee pain and symptoms, function, sports, and recreational activities) and when comparing between 82.30 kg and 95.00 kg and 95.00 kg or greater for WOMAC left knee pain and KOOS left knee pain and symptoms, and quality of life. We used a linear regression model that suggested as weight increased average WOMAC increased, β = 2.447, *P* < 0.001, 95% CI [1.790, 3.103] and average KOOS decreased, β = −2.739, *P* < 0.001, 95% CI [−3.625, −1.853]. See Figure 4. After adjusting for height, as weight increased and height decreased average WOMAC increased, for weight β = 2.856, *P* < 0.001, 95% CI [2.147, 3.564] and for height β = −1.078, *P* = 0.003, 95% CI [−1.788, −0.368] and KOOS decreased, for weight β = −3.094, *P* < 0.001, 95% CI [−4.051, −2.137] and for height β = 0.943, *P* = 0.056, 95% CI [−0.025, 1.910].

##### 3.2.2.2. Height

**Figure 5.**
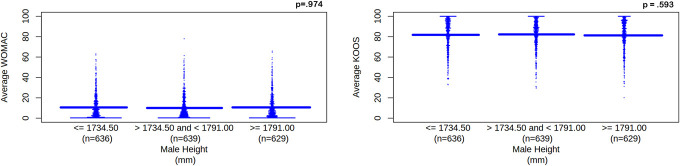
Average WOMAC and KOOS scores for male height category. Male height category is separated into tertiles. Height unit is mm, which represents millimeters. WOMAC and KOOS scores are unitless. WOMAC represents Western Ontario and McMaster Universities Osteoarthritis Index. As height increases, increasing WOMAC scores (left pane) represent worsening knee pain, disability, function, and total for left and right knees. KOOS represents Knee Outcomes of Osteoarthritis Scale. As height increases, decreasing KOOS scores (right pane) represent worsening knee pain and symptoms for left and right knees; worsening function, recreation, and daily activities; and worsening quality of life. Solid blue line represents the mean for each tertile. KOOS, Knee Outcomes of Osteoarthritis Scale; WOMAC, Western Ontario and McMaster Universities Osteoarthritis Index.

Height did not correlate with any WOMAC or KOOS scores. There were no significant differences (*P* > 0.05) when comparing height categories. Running the regression model confirmed that height change neither increased nor decreased average WOMAC or KOOS, β = 0.011, *P* = 0.974, 95% CI [−0.656, 0.678] and β = −0.247, *P* = 0.593, 95% CI [−1.153, 0.659]. See Figure 5.

##### 3.2.2.3. Waist circumference

**Figure 6.**
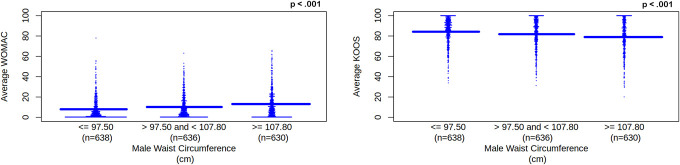
Average WOMAC and KOOS scores for male waist circumference category. Male waist circumference category is separated into tertiles. Waist circumference unit is cm, which represents centimeters. WOMAC and KOOS scores are unitless. WOMAC represents Western Ontario and McMaster Universities Osteoarthritis Index. As waist circumference increases, increasing WOMAC scores (left pane) represent worsening knee pain, disability, function, and total for left and right knees. KOOS represents Knee Outcomes of Osteoarthritis Scale. As waist circumference increases, decreasing KOOS scores (right pane) represent worsening knee pain and symptoms for left and right knees; worsening function, recreation, and daily activities; and worsening quality of life. Solid blue line represents the mean for each tertile. KOOS, Knee Outcomes of Osteoarthritis Scale; WOMAC, Western Ontario and McMaster Universities Osteoarthritis Index.

Waist circumference significantly correlated with all WOMAC and KOOS scores (*P*'s < 0.001). There were significant differences (*P* < 0.05) among waist circumference categories except when comparing 97.50 cm or less and between 97.50 cm and 107.80 cm (WOMAC right knee pain, stiffness, and disability and KOOS right knee pain and symptoms and quality of life), 107.80 cm or greater and between 97.50 cm and 107.80 cm (WOMAC left knee pain and KOOS right knee symptoms, left knee pain and symptoms). We used a linear regression model that suggested as waist circumference increased average WOMAC increased, β = 2.567, *P* < 0.001, 95% CI [1.912, 3.223] and average KOOS decreased, β = −2.618, *P* < 0.001, 95% CI [−3.509, −1.729]. See Figure 6.

## 4. Discussion

Our primary goal was to determine which anthropometric measure—weight, height, or waist circumference—was the strongest predictor of KOA (eg, pain, disability, and stiffness). When considering participants collectively and participants grouped by sex, we expected weight and waist circumference to be significantly associated with KOA—with waist circumference having a greater association than weight. We did not expect height to be associated with KOA.

We separated data by sex and grouped each anthropometric measure into tertiles. Tertiles ranged from lowest weight, height, and waist circumference to highest weight, height, and waist circumference. We expected comparisons by sex would demonstrate that the third tertile in (1) weight and (2) waist circumference groups would experience the greatest KOA discomforts with the waist circumference group feeling significantly more KOA discomforts than the weight group.

On average, there was a significant difference (*P* ≤ 0.05) between anthropometric measures and experiences of KOA. Female participants' experience of KOA averaged more than 20% (left knee stiffness: ∼22%, right knee pain: ∼26%, and left knee disability: ∼28%) worse than male participants. Previous studies indicated that female adults when compared to male adults experienced (1) radiographic osteoarthritis severity,^12,18^ (2) increased pain visual analog scale scores,^19^ and (3) KOOS pain decreases.^20,26^ There was not a statistical difference among participants between height and difficulties with daily activities (*P* = 0.134) nor height and knee-related quality of life (*P* = 0.107). Knee-related quality of life assessed perceptions regarding cognizance of knee discomforts, adjustments to lifestyle to accommodate knee and not cause further damage, confidence in stability of knee, and amount of difficulty with knee.^5^ Participants' quality of life views (average of 66.36%) were the worst among WOMAC and KOOS scores.

The National Health Statistics Report (NHSR) included average weight, height, and waist circumference measures for female and male adults.^27^ Average weight for female and male adults was 77.4 kg and 89.8 kg, respectively. Average height for female and male adults was 1617 mm and 1754 mm, respectively. Average waist circumference for female and male adults was 98.0 cm and 102.1 cm, respectively. The second tertile in this study for weight, height, and waist circumference included the NHSR average weight, height, and waist circumference.

In the weight category for each sex, WOMAC scores increased as weight increased with more increases for disability and KOOS scores decreased as weight decreased with more decreases for daily activities and quality of life. The increase in WOMAC scores and decrease in KOOS scores were expected because WOMAC scores increase with worse pain, while KOOS scores decrease with worse pain. Knee disability was higher than pain and stiffness, with complaints of stiffness being the lowest. Right knees experienced worse pain than left knees, which could be explained by the right-footedness trait of more than 60% of the population.^21^ Knee Outcomes of Osteoarthritis Scale quality of life scores were lower than pain, symptoms, and challenges with daily activities, which suggested that health, comfort, and happiness were inextricably intertwined and exacerbated by knee discomforts.

For height categories, female and male WOMAC and KOOS were approximately equal among tertiles. Participants in the second tertile experienced less discomfort from knee pain, stiffness, and disability than the participants in the first and third tertiles. The third tertiles' disability scores were higher than the first tertiles' disability scores, and disability scores were higher than pain and stiffness scores for female and male participants. Knee Outcomes of Osteoarthritis Scale quality of life scores were the lowest with the second tertiles slightly higher (better) than the first and third tertiles. We hypothesized that height would not be associated with KOA but were surprised that the second tertiles consistently experienced less osteoarthritis discomforts than the first tertile. Longer bones may place greater levels of stress on the joints, while shorter bones may make joints more susceptible to damage accounting for individuals in the first and third height tertiles.^2^

Female and male participants in the waist circumference categories experienced, to a lesser degree than the same participants in the weight categories, increasing WOMAC knee pain and stiffness. Western Ontario and McMaster Universities Osteoarthritis Index disability increased as waist circumference increased. Knee Outcomes of Osteoarthritis Scale left knee symptoms scores were the highest among KOOS scores indicating the least amount of discomfort. Unlike female and male weight categories and male waist circumference categories, KOOS activities' scores converged with KOOS quality of life scores for the female second and third waist circumference categories.

Female participants in the third tertile weight category reported worse WOMAC discomforts than participants in the third tertile waist circumference category by at least 8.5%, and in the first tertile waist circumference category, female participants reported worse discomforts than the first tertile weight category by at least 5.5%. Knee osteoarthritis discomforts from KOOS scores were less than 2% different between the third and first tertiles with the exception of KOOS daily living, sports, recreational activities, and quality of life, which were at least 3.4% worse when the third and first tertiles of weight and waist circumference categories were compared. Notably, there was a 24.2% and 21.75% chance that higher weight increases average WOMAC discomforts and KOOS discomforts, respectively, compared to a 15.95% and 12.83% chance that wider waist circumference increases average WOMAC discomforts and KOOS discomforts, respectively.

For male participants, KOA discomforts for the first tertile weight categories were <2% worse than the first tertile waist circumference categories for WOMAC and KOOS scores with the exception of left knee disability. Surprisingly, KOA discomforts between the third tertile weight and third tertile waist circumference categories were not greater than 1.5%. Knee osteoarthritis discomforts for second tertiles of weight and waist circumference were almost equivalent for WOMAC and KOOS reports. Interestingly, there was a 14.75% and 11.3% chance that higher weight increases average WOMAC discomforts and KOOS discomforts, respectively, compared to a 15.45% and 10.6% chance that wider waist circumference increases average WOMAC discomforts and KOOS discomforts, respectively.

Positive attributes of this study included the sample size of 4,602 participants from 5 states within the United States of America. In previous KOA studies prior to March 2020, the number of study participants with KOA did not exceed 4,000 participants for 90% of the studies.^3^ Participant waist circumference was collected for our large sample size. Tertiles for weight, height, and waist circumference were approximately equal, which improved the statistical power of the analysis tests used to compare the groups.

### 4.1. Limitations

One shortcoming of our study was that the quantity of female and male participants was not equal with female participants accounting for 58.6% of the participants. Provider utilization is significantly associated with sex and age (older women tend to seek professional care to treat health ailments more than men and young adults).^2,28^ Another shortcoming was that our dataset did not conform to national averages for weight, height, and waist circumference categories. We excluded participants with knee replacements or severe narrow joint spacing comorbid with KOA.

### 4.2. Conclusions and future directions

Weight and waist circumference predicted increases in KOA discomforts with weight being a much stronger predictor than waist circumference for female participants, while for male participants waist circumference and weight appeared to be equal predictors. Height increases did not predict KOA discomforts with the exception of female KOOS symptoms and quality of life. Knee pain, stiffness, symptoms, disability, function, and quality of life discomforts for the second tertile weight and waist circumference categories (female and male) were almost equivalent. Quality of life scores for both sexes were low with female participants reporting worse quality of life scores compared to male participants.

Recommendations for mitigating knee discomforts would be to lose weight and/or reduce waist circumference. Because (1) losing weight and keeping it off and (2) reducing waist circumference and keeping it reduced is difficult, our group is currently investigating how we can change pain perception by neuroplastic techniques to mitigate knee discomforts. It is our hope that adults can use these tools to reduce knee discomfort and in turn improve their quality of life.

## Disclosures

The authors have no conflict of interest to declare.

## Appendix A. Supplemental digital content

Supplemental digital content associated with this article can be found online at http://links.lww.com/PR9/A245.
