# Supplementary material for: Weight, height, waist circumference: association with knee osteoarthritis findings from the osteoarthritis initiative
Source: Pain Rep. 2024 Sep 20;9(5):e1187. doi: 10.1097/PR9.0000000000001187 (PMC11419520; doi:10.1097/PR9.0000000000001187)
Supplement: Supplementary file 1 [file painreports-9-e1187-s001.pdf]

**Appendix**
**List of Variables**

| Name | Label | Values |
|---|---|---|
| V00AGE | Age (years) | 45 - 79 |
| P02SEX | Sex | 1:male, 2:female, R:refused |
| P02RACE | racial background | 0:other,1: White or Caucasian,2: Black or African American,3: Asian, D: don't know/unknown/uncertain, R: refused |
| V00EDCV | Education | 0: less than high school graduate,1: high school graduate, 2: some college, 3: college graduate, 4: some graduate school, 5: graduate degree, M: missing, R: refused |
| V00CEMPLOY | employment status | 1: works for pay, 2: unpaid work for family business, 3: not working in part due to health, 4: not working other reasons, D: don't know/unknown/uncertain, M: missing, R: refused |
| V00INCOME | Income | 1: less than $10k, 2: $10k to < $25k, 3: $25k to < $50k, 4: $50k to < $100k, 5: $100k or greater, D: don't know/unknown/uncertain, M: missing, R: refused |
| V00MARITST | marital status | 1: Married, 2: Widowed, 3: Divorced, 4: Separated, 5: Never married, M: Missing, R: Refused |
| P01WEIGHT | weight (kg) | 42.60 - 135.50 |
| P0HEIGHT | height (mm) | 1475.50  - 1904.00 |
| V00ABCIRC | waist circumference (cm) | 62.80 - 149.30 |
| V00WOMKPR | WOMAC right knee pain score | 0 - 20 |
| V00WOMKPL | WOMAC left knee pain score | 0 - 20 |
| V00WOMSTFR | WOMAC right knee stiffness score | 0 - 8 |
| V00WOMSTFL | WOMAC left knee stiffness score | 0 - 8 |
| V00WOMADLR | WOMAC right knee disability score | 0 - 68 |
| V00WOMADLL | WOMAC left knee disability score | 0 - 68 |
| V00WOMTSR | WOMAC right knee total score | 0 - 96 |
| V00WOMTSL | WOMAC left knee total score | 0 - 96 |
| V00KOOSKPR | KOOS right knee pain score | 0 - 100 |

| | | |
|---|---|---|
| V00KOOSKPL | KOOS left knee pain score | 0 - 100 |
| V00KOOSYMR | KOOS right knee symptoms score | 0 - 100 |
| V00KOOSYML | KOOS left knee symptoms score | 0 - 100 |
| V00KOOSFSR | KOOS function, sports, and recreational score | 0 - 100 |
| V00KOOSQOL | KOOS quality of life score | 0 - 100 |

**Overall WOMAC and KOOS scores by weight, height, and waist circumference**

| | | | | | Confidence Interval | |
|---|---|---|---|---|---|---|
| | **Mean ± SD** | **n** | **R** | **p** | **Lower** | **upper** |
| **Weight** | | | | | | |
| Right knee: WOMAC Pain | 2.55 ± 3.26 | 4599 | 0.148 | < 0.001 | 0.12 | 0.176 |
| Right knee: WOMAC Stiffness | 1.63 ± 1.63 | 4601 | 0.122 | < 0.001 | 0.094 | 0.151 |
| Right knee: WOMAC Disability | 8.17 ± 10.59 | 4585 | 0.167 | < 0.001 | 0.139 | 0.195 |
| Right knee: WOMAC Total | 12.32 ± 14.68 | 4581 | 0.168 | < 0.001 | 0.139 | 0.196 |
| Left knee: WOMAC Pain | 2.37 ± 3.42 | 4602 | 0.154 | < 0.001 | 0.126 | 0.183 |
| Left knee: WOMAC Stiffness | 1.44 ± 1.66 | 4597 | 0.146 | < 0.001 | 0.117 | 0.174 |
| Left knee: WOMAC Disability | 8.29 ± 11.39 | 4579 | 0.169 | < 0.001 | 0.14 | 0.197 |
| Left knee: WOMAC Total | 12.08 ± 15.85 | 4574 | 0.17 | < 0.001 | 0.142 | 0.198 |
| Right knee: KOOS Pain | 83.68 ± 17.35 | 4599 | -0.144 | < 0.001 | -0.172 | -0.116 |
| Right knee: KOOS Symptoms | 86.06 ± 14.54 | 4602 | -0.116 | < 0.001 | -0.144 | -0.087 |
| Left knee: KOOS Pain | 84.95 ± 18.21 | 4600 | -0.154 | < 0.001 | -0.182 | -0.126 |
| Left knee: KOOS Symptoms | 86.63 ± 15.82 | 4602 | -0.14 | < 0.001 | -0.168 | -0.112 |
| KOOS Function, Sports, and Recreational Activities | 72.00 ± 25.88 | 3423 | -0.175 | < 0.001 | -0.207 | -0.142 |

| | | | | | | |
|---|---|---|---|---|---|---|
| KOOS Quality of Life | 66.36±22.44 | 4601 | -0.191 | < 0.001 | -0.219 | -0.163 |
| **Height** | | | | | | |
| Right knee: WOMAC Pain | 2.55 ± 3.26 | 4599 | -0.057 | < 0.001 | -0.085 | -0.028 |
| Right knee: WOMAC Stiffness | 1.63 ± 1.63 | 4601 | -0.071 | < 0.001 | -0.099 | -0.042 |
| Right knee: WOMAC Disability | 8.17 ± 10.59 | 4585 | -0.075 | < 0.001 | -0.103 | -0.046 |
| Right knee: WOMAC Total | 12.32 ± 14.68 | 4581 | -0.074 | < 0.001 | -0.103 | -0.045 |
| Left knee: WOMAC Pain | 2.37 ± 3.42 | 4602 | -0.056 | < 0.001 | -0.085 | -0.027 |
| Left knee: WOMAC Stiffness | 1.44 ± 1.66 | 4597 | -0.051 | < 0.001 | -0.08 | -0.023 |
| Left knee: WOMAC Disability | 8.29 ± 11.39 | 4579 | -0.061 | < 0.001 | -0.09 | -0.032 |
| Left knee: WOMAC Total | 12.08 ± 15.85 | 4574 | -0.062 | < 0.001 | -0.091 | -0.033 |
| Right knee: KOOS Pain | 83.68 ± 17.35 | 4599 | 0.038 | 0.011 | 0.009 | 0.066 |
| Right knee: KOOS Symptoms | 86.06 ± 14.54 | 4602 | 0.054 | <.001 | 0.025 | 0.083 |
| Left knee: KOOS Pain | 84.95 ± 18.21 | 4600 | 0.037 | 0.013 | 0.008 | 0.066 |
| Left knee: KOOS Symptoms | 86.63 ± 15.82 | 4602 | 0.036 | 0.014 | 0.008 | 0.065 |
| KOOS Function, Sports, and Recreational Activities | 72.00 ± 25.88 | 3423 | 0.026 | 0.134 | -0.008 | 0.059 |
| KOOS Quality of Life | 66.36±22.44 | 4601 | -0.024 | 0.107 | -0.053 | 0.005 |
| **Waist Circumference** | | | | | | |
| Right knee: WOMAC Pain | 2.55 ± 3.26 | 4599 | 0.135 | < 0.001 | 0.107 | 0.163 |
| Right knee: WOMAC Stiffness | 1.63 ± 1.63 | 4601 | 0.14 | <.001 | 0.111 | 0.168 |
| Right knee: WOMAC Disability | 8.17 ± 10.59 | 4585 | 0.183 | < 0.001 | 0.155 | 0.211 |
| Right knee: WOMAC Total | 12.32 ± 14.68 | 4581 | 0.178 | < 0.001 | 0.15 | 0.206 |
| Left knee: WOMAC Pain | 2.37 ± 3.42 | 4602 | 0.138 | < 0.001 | 0.11 | 0.166 |
| Left knee: WOMAC Stiffness | 1.44 ± 1.66 | 4597 | 0.145 | < 0.001 | 0.117 | 0.174 |

| | | | | | | |
|---|---|---|---|---|---|---|
| Left knee: WOMAC Disability | 8.29 ± 11.39 | 4579 | 0.176 | <.001 | 0.148 | 0.204 |
| Left knee: WOMAC Total | 12.08 ± 15.85 | 4574 | 0.173 | < 0.001 | 0.145 | 0.201 |
| Right knee: KOOS Pain | 83.68 ± 17.35 | 4599 | -0.124 | < 0.001 | -0.153 | -0.096 |
| Right knee: KOOS Symptoms | 86.06 ± 14.54 | 4602 | -0.115 | < 0.001 | -0.144 | -0.087 |
| Left knee: KOOS Pain | 84.95 ± 18.21 | 4600 | -0.131 | < 0.001 | -0.159 | -0.103 |
| Left knee: KOOS Symptoms | 86.63 ± 15.82 | 4602 | -0.125 | < 0.001 | -0.153 | -0.096 |
| KOOS Function, Sports, and Recreational Activities | 72.00 ± 25.88 | 3423 | -0.156 | < 0.001 | -0.188 | -0.123 |
| KOOS Quality of Life | 66.36±22.44 | 4601 | -0.143 | < 0.001 | -0.172 | -0.115 |

WOMAC represents Western Ontario and McMaster Universities Osteoarthritis Index, KOOS represents Knee Outcomes of Osteoarthritis Scale, SD represents standard deviation, n represents sample size, R represents Pearson coefficient, and p represents probability for continuous variables.
